# Supplementary material for: Various Anti-HSPA2 Antibodies Yield Different Results in Studies on Cancer-Related Functions of Heat Shock Protein A2
Source: Int J Mol Sci. 2020 Jun 16;21(12):4296. doi: 10.3390/ijms21124296 (PMC7352356; doi:10.3390/ijms21124296)
Supplement: Supplementary file 1 [file ijms-21-04296-s001.pdf]

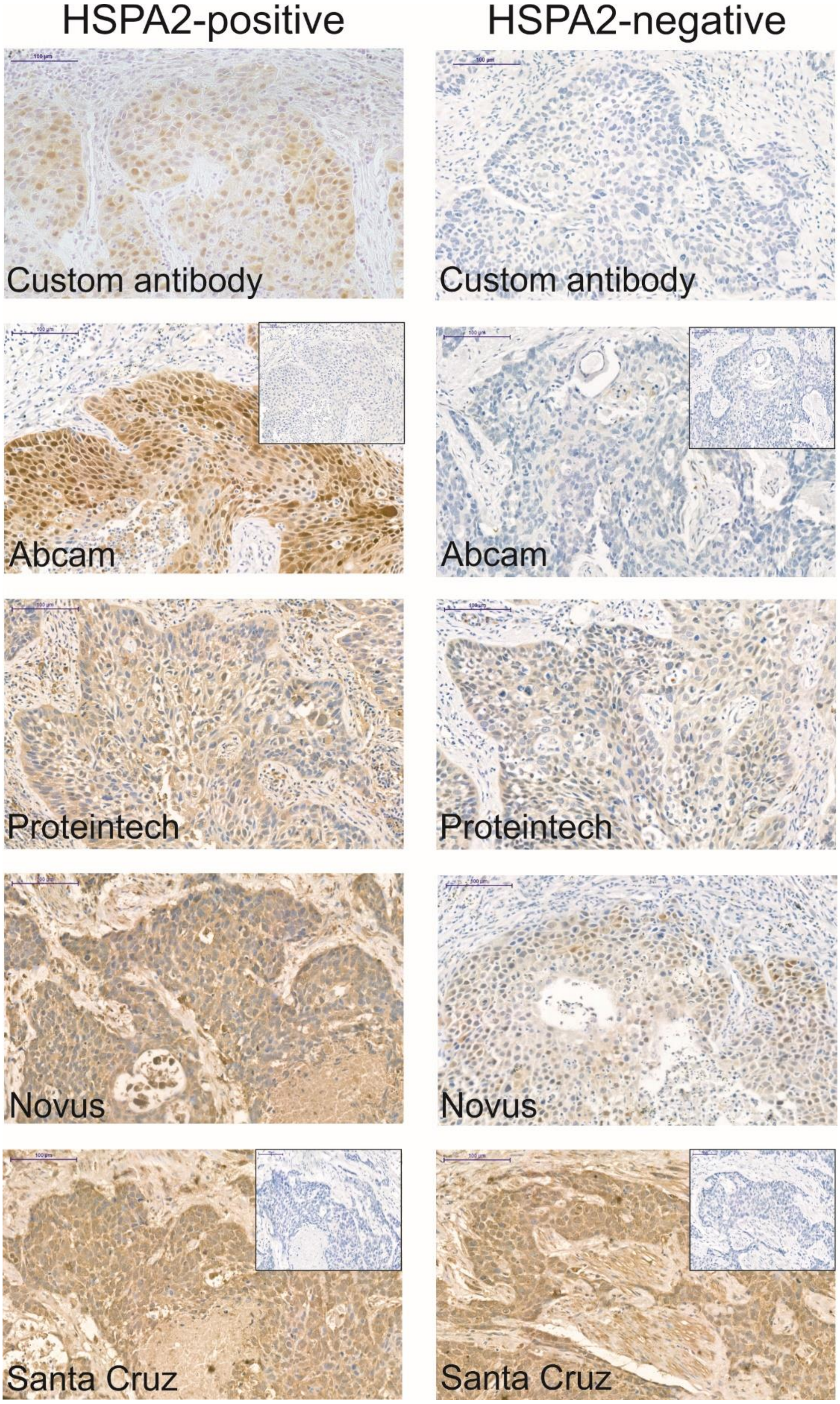

Figure S1. Results of immunostaining in NSCLC samples using specific and validated custom anti-HSPA2 antibody and commercially available anti-HSPA2 antibodies. Tissue specimens, after citric acid antigen retrieval were incubated with primary antibody overnight at 4°C. The details on antibodies are collected in Table 2.

Table S1. Results of in vitro studies on HSPA2 impact on cancer cells phenotype.

Tumor	Cell Lines	Method / Antibody	Phenotype of HSPA2-Deficient Cells	Ref.
cervical AD<br>BRCA	HeLa,<br>MCF 7	siRNA-mediated knockdown, TR / custom made	Senescent-like morphology, reduced metabolic activity, growth arrest.	[1]
cervical AD<br>BRCA	HeLa,<br>MCF7	siRNA-mediated knockdown, TR / custom made	Induction of apoptosis-like cell death, apoptosis-like chromatin condensation, reduction of LEDGF activity.	[2]
BLC	HTB-2, HTB-9, HTB-1, UMUC-3	shRNA-mediated knockdown, TR / custom made	Reduced proliferation, clone forming ability and invading potential (HTB-1, UMUC-3), reduced motility (HTB-1).	[3]
cervical<br>SCC<br>AD	SiHa, CaSki,<br>C-33 A<br>HeLa	shRNA-mediated knockdown, TR / custom made	Reduced proliferation, clone forming ability, motility (SiHa, CaSki), invading potential.	[4]
renal cell carcinoma	A704, ACHN, Caki-1	shRNA-mediated knockdown, TR / custom made	Reduced proliferation, metabolic activity, clone forming ability, motility, invading potential (A704, Caki-1).	[5]
colorectal cancer	COLO205, HCT116	shRNA-mediated knockdown, TR / custom made	Reduction in proliferation, metabolic activity, clone forming ability, motility, invading potential.	[6]
BRCA	MCF7, MDA-MB-231	shRNA-mediated knockdown, TR / custom made	Induction of spontaneous apoptosis, switch to senescence. Reduced proliferation, metabolic activity, clone forming ability, motility, invading potential.	[7]
OV<br><br>ovary AD	A-10<br><br>Caov-3, SKOV3	shRNA-mediated knockdown, / custom made	Induction of spontaneous apoptosis, switch to senescence. Reduced proliferation, metabolic activity, clone forming ability, motility, invading potential.	[8]
NSCLC	NCI-H23, NCI-H1299	shRNA-mediated knockdown, stable / Abcam EPR4596	No effect on growth, metabolic activity, clone forming ability, resistance to platinum derivatives and bortezomib. Similar phenotype showed cells deficient in HSPA1. Pan-HSPA inhibition reduced metabolic activity and activated cell death.	[9]
lung AD	A549, NCI-H1975	siRNA-mediated knockdown / no data	Reduced proliferation, G1/S cell cycle arrest mediated in part via activation of the Erk1/2 pathway and probably IRE1α/PERK-mediated endoplasmic reticulum stress.	[10]

Abbreviations: AD, adenocarcinoma; BLC, Bladder Urothelial Carcinoma; BRCA, Breast invasive carcinoma; OV, ovarian serous cystadenocarcinoma; NSCLC, non-small cell lung carcinoma; SCC, squamous cell carcinoma; TR, transient

References:

1. Rohde, M.; Daugaard, M.; Jensen, M.H.; Helin, K.; Nylandsted, J.; Jäättelä, M. Members of the heat-shock protein 70 family promote cancer cell growth by distinct mechanisms. *Genes Dev.* **2005**, *19*, 570-582.
2. Daugaard, M.; Kirkegaard-Sørensen, T.; Ostenfeld, M.S.; Aaboe, M.; Høyer-Hansen, M.; Orntoft, T.F.; Rohde, M.; Jäättelä, M. Lens epithelium-derived growth factor is an Hsp70-2 regulated guardian of lysosomal stability in human cancer. *Cancer Res.* **2007**, *67*, 2559-2567.

3. Garg, M.; Kanojia, D.; Saini, S.; Suri, S.; Gupta, A.; Surolia, A.; Suri, A. Germ cell-specific heat shock protein 70-2 is expressed in cervical carcinoma and is involved in the growth, migration, and invasion of cervical cells. *Cancer* **2010a**, *116*, 3785-3796.
4. Garg, M.; Kanojia, D.; Seth, A.; Kumar, R.; Gupta, A.; Surolia, A.; Suri, A. Heat-shock protein 70-2 (HSP70-2) expression in bladder urothelial carcinoma is associated with tumour progression and promotes migration and invasion. *Eur. J. Cancer* **2010b**, *46***,** 207-215.
5. Singh, S.; Suri, A. Targeting the testis-specific heat-shock protein 70-2 (HSP70-2) reduces cellular growth, migration, and invasion in renal cell carcinoma cells. *Tumour Biol.* **2014**, *35*, 12695-12706.
6. Jagadish, N.; Agarwal, S.; Gupta, N.; Fatima, R.; Devi, S.; Kumar, V.; Suri, V.; Kumar, R.; Suri, V.; Sadasukhi, T.C.; Gupta, A.; Ansari, A.S.; Lohiya, N.K.; Suri, A. Heat shock protein 70-2 (HSP70-2) overexpression in breast cancer. *J. Exp. Clin. Cancer Res.* **2016b**, *35*, 150.
7. Jagadish, N.; Parashar, D.; Gupta, N.; Agarwal, S.; Suri, V.; Kumar, R.; Suri, V.; Sadasukhi, T.C.; Gupta, A.; Ansari, A.S.; Lohiya, N.K.; Suri, A. Heat shock protein 70-2 (HSP70-2) is a novel therapeutic target for colorectal cancer and is associated with tumor growth. *BMC Cancer* **2016a**, *16*, 561.
8. Gupta, N.; Jagadish, N.; Surolia, A.; Suri A. Heat shock protein 70-2 (HSP70-2) a novel cancer testis antigen that promotes growth of ovarian cancer. *Am. J. Cancer Res.* **2017**, *7*, 1252-1269.
9. Sojka, D.R.; Gogler-Pigłowska, A.; Vydra, N.; Cortez, A.J.; Filipczak, P.T.; Krawczyk, Z.; Scieglinska, D. Functional redundancy of HSPA1, HSPA2 and other HSPA proteins in non-small cell lung carcinoma (NSCLC); an implication for NSCLC treatment. *Sci. Rep*. **2019**, *9*, 14394.
10. Cao, L.; Yuan, X.; Bao, F.; Lv, W.; He, Z.; Tang, J.; Han, J.; Hu, J. Downregulation of HSPA2 inhibits proliferation via ERK1/2 pathway and endoplasmic reticular stress in lung adenocarcinoma. *Ann. Transl. Med.* **2019**, *7*, 540.
